# Metabolomic Evidence for a Field Effect in Histologically Normal and Metaplastic Tissues in Patients with Esophageal Adenocarcinoma^1^

**DOI:** 10.1016/j.neo.2016.11.003

**Published:** 2017-01-31

**Authors:** Michelle A.C. Reed, Rishi Singhal, Christian Ludwig, John B. Carrigan, Douglas G. Ward, Phillipe Taniere, Derek Alderson, Ulrich L. Günther

**Affiliations:** * Institute of Cancer and Genomic Sciences, University of Birmingham, Birmingham, B15 2TT, UK; † Queen Elizabeth Hospital, Edgbaston, Birmingham, B15 2TH, UK

**Keywords:** EAC, esophageal adenocarcinoma, BO, Barrett's esophagus, HGD, high-grade dysplasia, NMR, nuclear magnetic resonance, PCA, principle component analysis, PLS-DA, partial least squares discriminant analysis, ML-PLS-DA, multilevel partial least squares discriminant analysis, AUROC, area under the receiver operator curve

## Abstract

Patients with Barrett's esophagus (BO) are at increased risk of developing esophageal adenocarcinoma (EAC). Most Barrett's patients, however, do not develop EAC, and there is a need for markers that can identify those most at risk. This study aimed to see if a metabolic signature associated with the development of EAC existed. For this, tissue extracts from patients with EAC, BO, and normal esophagus were analyzed using ^1^H nuclear magnetic resonance. Where possible, adjacent histologically normal tissues were sampled in those with EAC and BO. The study included 46 patients with EAC, 7 patients with BO, and 68 controls who underwent endoscopy for dyspeptic symptoms with normal appearances. Within the cancer cohort, 9 patients had nonneoplastic Barrett's adjacent to the cancer suitable for biopsy. It was possible to distinguish between histologically normal, BO, and EAC tissue in EAC patients [area under the receiver operator curve (AUROC) 1.00, 0.86, and 0.91] and between histologically benign BO in the presence and absence of EAC (AUROC 0.79). In both these cases, sample numbers limited the power of the models. Comparison of histologically normal tissue proximal to EAC versus that from controls (AUROC 1.00) suggests a strong field effect which may develop prior to overt EAC and hence be useful for identifying patients at high risk of developing EAC. Excellent sensitivity and specificity were found for this model to distinguish histologically normal squamous esophageal mucosa in EAC patients and healthy controls, with 8 metabolites being very significantly altered. This may have potential diagnostic value if a molecular signature can detect tissue from which neoplasms subsequently arise.

## Introduction

In many Western countries, rates of esophageal adenocarcinoma (EAC) have been increasing for more than 20 years, particularly among overweight, white men and those with severe gastroesophageal reflux disease [1], [2]. Among patients with gastroesophageal reflux disease, some develop Barrett's esophagus (BO), characterized by metaplastic columnar epithelium in which mucus-secreting goblet cells appear. In some patients, this lining becomes unstable, progressing from low-grade to high-grade dysplasia (HGD) and then neoplasia. Identification of this at-risk population presently relies on endoscopic surveillance of large cohorts of patients with BO, most of whom will not develop a cancer.

The exact risk of patients with BO and HGD developing EAC is not known, but one meta-analysis gave a weighted incidence rate of 6.58 per 100 patient-years during the first 1.5 to 7 years [3]. Likewise, Konda et al. suggested that the true rate of invasive EAC was 12% in patients diagnosed with HGD who underwent surgical resection. The other 88% of patients had only HGD or intramucosal carcinoma, potentially treatable by endoscopic ablation or endoscopic mucosal resection [4], [5]. New markers are needed to distinguish BO patients at highest risk of developing EAC and to guide treatment options [6]. Identifying patients with BO and progression to HGD based on histology alone can be challenging because of sampling limitations and interobserver variability among pathologists [7]. In addition, most endoscopic studies have focused on the Barrett's epithelium itself, with little attention given to the squamous epithelium.

The presence of genetic mutations and evidence of dysregulation in histologically unaffected tissues adjacent to cancers implies a “field effect” that might be exploited if signatures exist that are associated with progression to HGD and intramucosal cancer in BO patients. [8]. Many different field effect biomarkers including changes in gene and protein expression, and epigenetic and metabolomic markers have been reported for different types of cancers [9]. Different techniques have been used to detect field effects in EAC, including nanoscale structural properties [10], [11] and nuclear magnetic resonance (NMR)–based metabolomics of histologically normal cells proximal to EAC [12].

Some of the previous EAC metabolomics studies were based on different types of samples, relying on serum or urine samples to separate EAC patients from normal or other cancer patients. Sanchez-Espiridian et al. identified a panel of possible serum biomarkers to distinguish EAC patients and healthy controls using a liquid chromatography/mass spectrometry (MS) apprEACh for samples from more than 650 patients and controls [13]. Likewise, Davis et al. used ^1^H-NMR metabolomics on urine samples to distinguish EAC patients or Barrett's patients from controls. Ikeda et al. used gas chromatography/MS metabolomics on human serum to identify various different biomarkers that distinguished EAC patients from colon cancer patients, gastric cancer patients, and controls [14]. Zhang et al. used liquid chromatography/MS and NMR to build a model based on samples from cancer patients and controls to address the more challenging task of separating EAC patients from patients with BO and HGD using serum metabolite levels [15].

There have also been two tissue metabolomics studies on EAC. Yakoub et al. [12] reported that a high phosphocholine/glutamate ratio indicated the presence of cancer proximal to histologically normal tissue in a ^1^H Magic Angle Spinning NMR study of 35 EAC patients and 52 controls. Doran et al. observed a decrease in the ratio of carbohydrate to creatine-containing metabolites in Barrett's tissue samples in the presence of EAC compared with Barrett's in the absence of EAC for 29 controls and 43 cancer patients [16].

The present investigation was carried out using ^1^H-NMR spectroscopy–based metabolomics looking at tissue samples from patients with EAC, patients with BO, or controls. The same samples were also subject to a previous Matrix-Assisted Laser Desorption/Ionisation analysis [17]. Metabolic profiling from both squamous and columnar epithelia across a range of patients was undertaken. The goal of this study was to identify specific metabolic profiles in EAC tissues compared to BO and control tissues, including metabolic changes in the histologically nonneoplastic tissues adjacent to EAC. This study attempted to identify metabolic markers that identify EAC and see if there was evidence of a field effect in histologically normal squamous or nondysplastic columnar epithelia in cancer patients.

## Material and Methods

### Tissue Samples

Tissue samples were obtained from patients with EAC, patients with BO, and controls (patients undergoing upper gastrointestinal endoscopy for dyspeptic symptoms but without endoscopic abnormalities) who presented to University Hospitals, Birmingham, UK, between May 2009 and March 2010. Overall ^1^H-NMR spectra for 211 polar extracts from tissue samples were used in this study (Table 1).

#### Ethics Approval and Consent to Participate

Patients were recruited from University Hospitals Birmingham between May 2009 and March 2010. All patients included in this study gave informed consent. Ethical approval for this study was obtained from South Birmingham Research Ethics Committee (REC reference number 08/H1207/3).

#### Sample Collection

Healthy normal esophageal squamous mucosa biopsies were obtained from 68 patients presenting with symptoms of benign gastroesophageal reflux disease (NN). A total of 51 EAC patients contributed samples either pre- or postchemotherapy (or both). Their disease was staged as T2/3N0/1. Five EAC patients with other major comorbidities were not included in the subsequent analysis. There were 7 Barrett's patients who contributed both histologically normal and Barrett's tissue samples.

For patients with gastroesophageal malignancies, biopsies of tumor mucosa; histologically normal tissue at least 5 cm from tumor; and, if available, Barrett's mucosa were obtained under general anesthetic prior to staging laparoscopy. For some patients, a second set of samples was collected after chemotherapy. All diagnoses were histologically confirmed using biopsies. For Barrett's patients, biopsies were obtained at the time of endoscopy for Barrett's mucosa and for normal mucosa at least 5 cm from the Barrett's mucosa. Samples from controls were also obtained at the time of endoscopy. All tissue vials were stored on ice for 1 hour and then at −80°C. Patient and sample data are summarized in Table 1.

#### Sample Preparation

Methanol chloroform extraction, as originally described by Bligh and Dwyer [18], was used to prepare polar extracts for NMR analysis. Tissues were homogenized using a Precellys 24 ceramic bead-based homogenizer (Stretton Scientific Ltd., UK). All solvents were kept on ice. Eight microliters per milligram of methanol and 2.5 μl/mg of water were added to each Precellys tube, and tubes were placed in the Precellys 24 homogenizer for two 10-second bursts at 6400 rpm. The homogenized mixture was pipetted into a clean 1.8-ml glass vial using a Pasteur pipette. Eight microliters per milligram of chloroform and 4 μl/mg of water were subsequently added to each vial. The vials were vortexed at full power for 30 seconds each and left on ice for 10 minutes. They were then centrifuged at 1800*g* (3000 rpm) at 4°C for 10 minutes. The polar fraction was dried in a centrifugal evaporator (SpeedVac).

### NMR Spectra

#### Data Acquisition

For NMR analysis, dried polar extracts were then resuspended in 100 mM sodium phosphate, pH 7, with 0.5 mM TSP as internal reference and 10% D2O as lock solvent. All ^1^H Nuclear Overhauser Spectroscopy spectra were acquired on a 600-MHz Bruker AVANCE2 spectrometer with a 1.7-mm TXI probe at 288 K using the standard Bruker sequence, noesygppr1d with a very short Nuclear Overhauser Spectroscopy mixing time of 10 milliseconds and with a 9.8-microsecond 1H hard pulse at 17 dB. A total of 32 k points were acquired over an acquisition time of 2.2 seconds, giving a spectral width of 7289 Hz. With an interscan delay of 4 seconds and 512 scans per sample, total experiment time was about 53 minutes per spectrum.

#### Data Processing

Free induction decays were zero-filled to 32 K points and multiplied by a squared cosine window function before Fourier transformation and phasing. All spectra were then aligned on the TSP signal, a spline baseline correction was applied, and the water (4.49-5.89 ppm) and TSP (below 0.14 ppm) regions of the spectra were excluded. A number of regions were subjected to segmental alignment using the icoshift software [19] to align resonances slightly shifted by small differences, for example, in sample pH. Finally, all spectra were scaled using total spectra area scaling. For multivariate analysis, a generalized logarithmic transformation (*y*_0_ = 1e-7, *λ* = 0.0005) was applied to increase the weighting given to less intense resonances [20]. Then, the *x*-block data were mean-centered, and principal component analysis (PCA) was performed initially to look for any model-free group separation and to identify spectra with high Q-residuals that should be excluded from subsequent analysis.

#### Statistical Analysis

For the partial least squares discriminant analysis (o-PLS-DA), the *x*-block data were mean-centered and subject to orthogonal signal correction. Cross-validation used Venetian blinds except in cases of 20 or fewer spectra in the model, in which case the “leave-one-out” method was used. The permutation test had *n* = 100 cycles. For multilevel (ML)-PLS-DA, double cross-validation was used with 20 repeats and a maximum of 3 latent variables, and then a 200-cycle permutation test was performed. For the PLS-DA models, the following statistical parameters are reported: the area under the receiver operator curve (AUROC), the cross-validated error rate (CVER), and the permutation test *P* values (*P*).

#### Sample Selection and Statistical Models

The specific statistical method and parameters used are summarized in Table S1. Pre- and postchemotherapy samples were available for some but not all of the patients. For most of the study, we used prechemotherapy samples and o-PLS-DA. However, for the smaller groups, we also included some postchemotherapy samples. Specifically, there were 30 and 29 histologically normal samples from EAC patients pre- and postchemotherapy. Fifteen of these were paired (i.e., from the same patients). Likewise, 28 and 29 EAC samples from EAC patients, pre- and postchemotherapy, were available. Fourteen of these were paired. Because approximately half of the samples were unpaired, o-PLS-DA was used rather than ML-PLS-DA. The only cases where ML-PLS-DA was used were comparisons between normal versus Barrett's tissue in Barrett's patients (classes 2 and 4) and a model where normal versus Barrett's in EAC patients and Barrett's versus EAC in EAC patients were compared.

#### Univariate Analyses

To compare metabolite concentrations, one well-resolved peak was picked for each metabolite in the first spectrum, and peaks were picked in the other spectra in an automated manner using in-house subroutines of MetaboLab [21]. The mean and standard deviations for that metabolite for different classes were calculated using Matlab functions mean and std., respectively. The Shapiro-Wilk test was used to test each class's metabolite intensities for normality (cutoff, *P* = .05). Depending upon the results of the normality test, one of four tests was performed. If the data were unpaired, then if the two Shapiro tests retained the null hypothesis, the Welch test was used; otherwise, the Wilcoxon rank sum test was used. If the data were paired, then if the Shapiro test retained the null hypothesis, the paired *t* test was used, but if the Shapiro test rejected the null hypothesis, the paired Wilcoxon signed rank test was used. In all cases, a 5% cutoff (*P* value < .05) was used to test the null hypothesis that the peak intensities for the two classes were the same.

## Results

**Figure 1:**
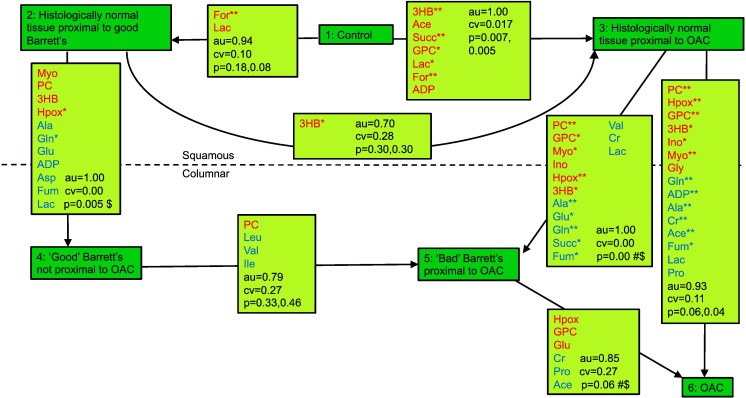
Overview of model with statistical data and metabolic changes between classes. PLS-DA models were assessed by cross-validation and permutation testing (au = area under receiver operator curve, cv = cross-validated error rate, p = permutation test *P* value). For individual metabolite changes, *P* values are reported: *P < .005, **P < .0005; red: metabolite level increased, blue: metabolite level decreased. $: a paired significance test was used. # refers to the 10-case model with paired samples from EAC patients, both either pre- or postchemotherapy.

Metabolite levels were analyzed in nine classes of tissue samples as described in Table 1. Multivariate models were run to compare different tissue classes to see where the most profound changes in the metabolome occurred. Individual metabolite levels were compared to determine which metabolite levels were statistically significantly altered between classes. The results of the multivariate models comparing different tissue classes to identify the most profound changes in the metabolome are summarized in Figure 1. Metabolite levels varied with tissue type, but there was also considerable intragroup variability (Figure S1). Table S1 summarizes the results for all class comparisons giving statistical parameters for the PLS-DA models and information about individual metabolite level changes.

### Effect of Chemotherapy (Class 6 vs Class 9 and Class 3 vs Class 7)

This initial test was important to clarify whether tissue samples from pre- and postchemotherapy patients could be treated as one similar group in subsequent models.

For the cancer tissue, PCA showed separation (between groups 6 and 9) (Figure S2*A*) and PLS-DA demonstrated near-perfect separation (Figure S2*B*) between the two groups (AUROC = 1.00, CVER = 0.018, *P* = .007, .007) (Figure S2*C*). The most discriminating metabolites with *P* < .00005 were lactate which increased and formate which decreased postchemotherapy.

The PCA model built for normal squamous epithelium from cancer patients (groups 3 vs 7) also showed good separation (Figure S2*D*), confirmed by the PLS-DA model (Figure S2*E*) between the pre- and postchemotherapy groups (AUROC = 1.00, CVER = 0.00, *P* = .005, .005) (Figure S2*F*). Here the metabolite level changes were often more statistically significant, with key metabolite changes being elevated valine, succinate, myoinositol, glycine, creatine, and lactate, and reduced formate and aspartate. The discriminating metabolites were very similar in histologically normal tissue to those seen in cancer tissue (Figure S3), suggesting that the observed metabolic signature reflects the general effect of chemotherapy rather than a cancer-specific response. These results imply that in looking for differences between tissues in controls, Barrett's patients, and cancer patients, it is essential to use only prechemotherapy samples from EAC patients. For these reasons, all except one of the subsequent comparative analyses relied on prechemotherapy samples only.

### Overview of Metabolome Changes between Different Tissue Types

Figure 1 summarizes the differences between tissue classes showing the strength of PLS-DA models in terms of their statistical significance and indicating which metabolites were statistically significantly altered between tissues. The figure is laid out to show squamous tissues at the top and columnar tissues at the bottom.

We expected the strongest metabolic changes between the histologically normal tissue in controls and the EAC cancer tissue in EAC patients. Hence, we first compared these tissues using a PLS-DA model. Then, we looked at models that involved tissues that are histologically different, e.g., comparing Barrett's (columnar) tissue in Barrett's patients with histologically normal (squamous) tissue in Barrett's patients to derive specific differences between squamous and metaplastic columnar tissues. Next, we looked at the models of greatest clinical relevance, namely, those that seek to differentiate different columnar tissues, e.g., Barrett's tissues in Barrett's and EAC patients. Finally, we compared histologically “normal tissues” from controls, Barrett's patients, and EAC patients to look for evidence of field effects in Barrett's and EAC patients.

### Controls versus Prechemotherapy EAC Tissue (Class 1 vs Class 6)

**Figure 2:**
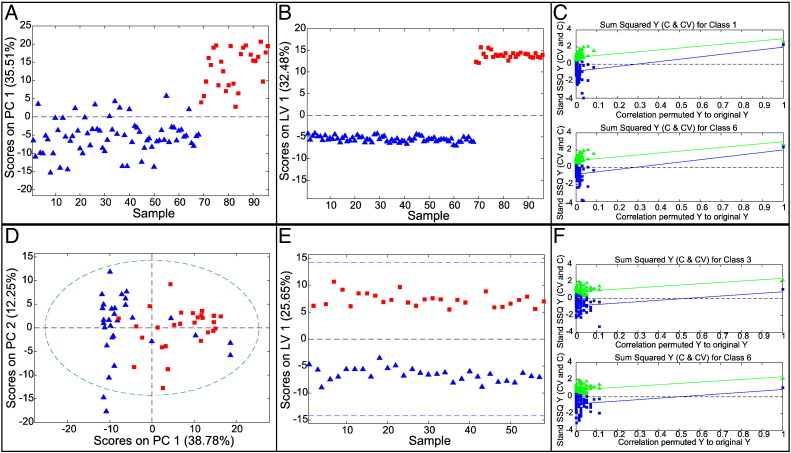
(A–C) Multivariate analysis of normal tissue in controls versus cancer tissue in EAC patients. (A) Scores plot for PCA model. (B) Scores plot for PLS-DA model. (C) Permutation test for *n* = 100. Normal tissue in controls as blue triangles; EAC tissue in cancer patients as red squares. (D–F) Multivariate analysis for normal tissue proximal to EAC and cancer tissue from EAC patients prechemotherapy. (D) Scores plot for PCA model. (E) Scores plot for PLS-DA model. Normal tissue proximal to EAC as blue triangles; EAC tissue as red rectangles. (F) Permutation test, *n* = 100.

In an initial PCA, one EAC spectrum gave a very high Q-residual and was removed. The subsequent PCA model had 68 control and 27 EAC spectra with good separation between the groups (Figure 2*A*). The two groups also showed excellent separation in PLS-DA (Figure 2*B*) confirmed by statistical analysis. For the PLS-DA model, AUROC was 1.00, with a CVER of 0.00 and permutation test *P* values of .008 and .005 (Figure 2*C*) for control and cancer tissue, respectively, in random *t* tests. This model demonstrated that cancer tissue has a significantly different metabolic signature compared with normal tissue. Many individual metabolite levels were significantly altered (*P* < .0005); specifically, myoinositol, inosine, hypoxanthine, 3-hydroxybutyrate, glycerophosphocholine, phosphocholine, and formate were all elevated, whereas glutamine, alanine, creatine, ADP, and fumarate were all reduced.

### Metabolic Differences between Squamous and Columnar Tissues (Class 4 vs Class 2, Class 5 vs Class 3, and Class 6 vs Class 3)

From Figure 1, it is clear that metabolomics PLS-DA models readily resolve histologically distinct tissues. For example, the PLS-DA model for class 4 versus class 2 has an AUROC of 1.00, a CVER of 0.00, and a *P* value of .005. The histological changes observed in the shift from squamous to columnar tissues are fully reflected in very strong metabolic signatures, both in the case of cancer patients and in those with histologically nondysplastic Barrett's.

### Histologically Normal Squamous and EAC Tissues from EAC Patients Prechemotherapy (Classes 3 vs 6)

A similar signature should also be expected if one compares histologically normal and EAC tissues, both from EAC patients, although more significant changes must be expected than for the previous model. Spectra from 30 “normal” and 28 cancer specimens were used. Following PCA analysis (Figure 2*D*), one cancer tissue spectrum was omitted because of high Q-residuals. The resulting PLS-DA model (Figure 2, *E*–*F*; AUROC = 0.93, CVER = 0.106, *P* = .058/.039) showed good group separation in latent variable 1 The differentiating metabolites were similar to those in the controls versus EAC model, but the fold changes were often reduced.

### Barrett's Tissue in the Presence and Absence of EAC (Class 5 vs 4)

**Figure 3:**
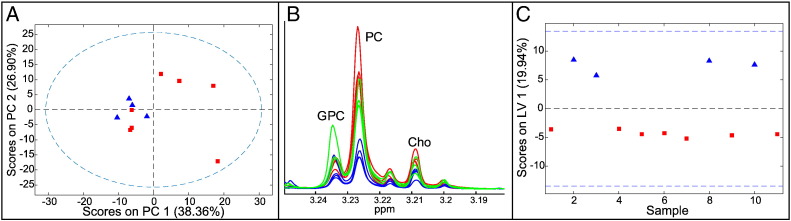
Multivariate analysis for Barrett's tissues in Barrett's and EAC patients. (A) Scores plot for PCA model (blue triangles: EAC patients, red squares: Barrett's patients); (B) choline region of the ^1^H-NMR spectrum (red: Barrett's from EAC patients, green: Barrett's from Barrett's patients that group in PCA scores with Barrett's from EAC patients, blue: Barrett's from Barrett's patients that do not group with Barrett's from EAC patients). (C) Scores plot for PLS-DA model (blue triangles: EAC patients, red squares: Barrett's patients).

Histologically nondysplastic Barrett's tissue samples were available from four EAC patients prechemotherapy. A PCA model (Figure 3*A*) showed that three of seven samples from patients with BO alone grouped with the four EAC-associated Barrett's samples, mainly because they had elevated phosphocholine and glycerophosphocholine (Figure 3*B*). A PLS-DA model (Figure 3*C*) gave a weak separation (CVER = 0.27, AUROC = 0.79) of these metaplastic tissues from Barrett's and EAC patients. The only statistically significantly altered metabolites between cancer-associated and cancer-free Barrett's samples were phosphocholine, which was elevated, and leucine, isoleucine, and valine, which were reduced in the EAC-associated Barrett's.

### Comparison of Histologically Normal, Barrett's, and EAC Tissues in Patients with EAC

**Figure 4:**
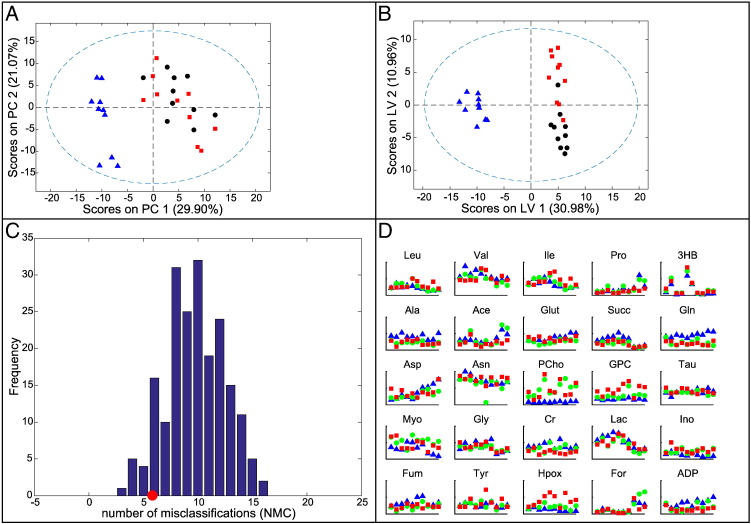
O-PLS-DA model for spectra from the three different tissue types from EAC patients: histologically normal tissue proximal to EAC tissue, Barrett's tissue proximal to EAC tissue, and EAC tissue. (A) Scores plot for PCA: normal (blue triangles), Barrett's (red squares), and cancer (black circles). (B) Scores plot for PLS-DA: normal (blue triangles), Barrett's (red squares), and cancer (black circles). (C) Permutation test for paired Barrett's and EAC samples. Red circle = number of misclassifications in model. Bar chart showing the number of misclassifications in each of 200 permutation tests, corresponding to a *P* value of .055. (D) Metabolite levels for these 10 cases; first 6 sets are postchemotherapy, and the last 4 sets are prechemotherapy.

An O-PLS-DA model was constructed to compare the three tissue types of histologically normal tissue, nondysplastic Barrett's tissue, and cancer tissue, all from cancer patients. Of the 51 EAC patients recruited overall, only 7 had all 3 tissue types available to be entered into this model. Of these, three patients had complete sets of the three tissue types available both prechemotherapy and postchemotherapy. A further four patients had a complete set of samples postchemotherapy but no samples prechemotherapy. Thus, there were just 10 sets of samples available. Although this model contains pre- and postchemotherapy samples, each class contains the same number of pre- and postchemotherapy samples. Therefore, the model should not be biased by the effect of chemotherapy (see Figure S4 for further details). PCA, shown in Figure 4*A*, separated the histologically normal squamous tissue from the columnar Barrett's and EAC tissues but not the Barrett's from the EAC tissue. The PLS-DA model (Figure 4*B*) showed good separation of the normal tissue from the columnar tissue in latent variable 1 and reasonable separation of the Barrett's and cancer tissue in latent variable 2 (AUROC 1/0.86/0.91; CVER = 0, 0.225, 0.125; and *P* = .021, .138, .049 for normal, Barrett's, and cancer tissues, respectively).

Considering that the signatures for BO and EAC tissue were similar, ML-PLS-DA was performed in an attempt to build a model that separated paired Barrett's and cancer tissue samples from the same patients. The resulting model still had a relatively high error rate (AUROC = 0.85, CVER = 0.27), but the permutation test result was .055, suggesting that the model had some value (Figure 4*C*). The following metabolites were statistically significantly altered (*P* < .05): glutamate, glycerophosphocholine, and hypoxanthine were increased, and propionate and creatine were decreased.

Interestingly, the only metabolites that were statistically significantly altered (in both paired and unpaired *t* tests) in a consistent direction from histologically normal tissue to Barrett's tissue to EAC tissue in EAC patients were glycerophosphocholine and hypoxanthine, which increased, and creatine, which decreased (Figure 4*D*).

### Comparisons of Histologically Normal Squamous Esophageal Mucosa in Controls, Barrett's Patients, and EAC Patients (Classes 1, 2, and 3)

Three-way and pairwise comparisons were carried out for classes 1, 2, and 3 to see whether changes in BO and EAC patients are observed in histologically normal tissue proximal to abnormal tissue.

#### Three-Way Comparison (Classes 1, 2, and 3)

**Figure 5:**
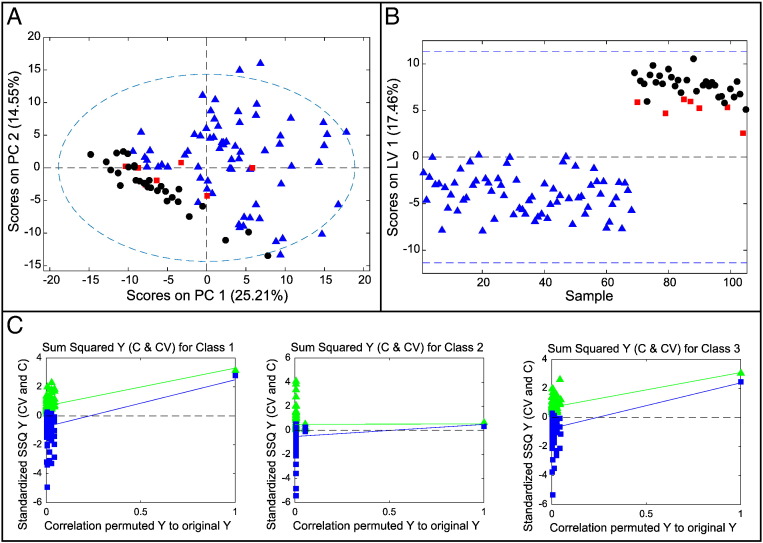
Multivariate models for histologically normal tissue from controls, Barrett's patients, and EAC patients (prechemotherapy). (A) Scores plot for PCA model. (B) Scores plot for PLS-DA model. (C) Permutation test with *n* = 100. Color coding in (A) and (B): Normal tissues from controls (blue triangles), from Barrett's patients (red squares), and from EAC patients (black circles).

An initial PCA model compared histologically normal tissue from controls (1), Barrett's patients (2), and EAC patients (3). This readily separated classes 1 and 3, with BO samples (2) overlapping the other two classes (Figure 5*A*). The corresponding PLS-DA model (Figure 5, *B* and *C*) readily separated class 1 well from 2 and 3 but was poor at distinguishing classes 2 and 3 (CVER = 0.028, 0.23,0.15; AUROC = 1.00, 0.73, 0.97; *P* = .005, .288, .005). The power of this model was limited by the very small number of samples from BO patients.

#### Histologically Normal Tissue from Controls and from BO Patients (Class 1 vs Class 2)

The quality of this PLS-DA model (AUROC = 0.94, CVER = 0.10, *P* = .08, .18) was limited by the small sample size of class 2. Metabolites contributing to this separation included formate which increased and 3-hydroxybutyrate which decreased in Barrett's patients.

#### Histologically Normal Tissue from BO and EAC Patients (Class 2 vs 3)

This PLS-DA model was worse than the previous model (AUROC = 0.70, CVER = 0.28, *P* = .30, .30), suggesting that the normal tissue in Barrett's patients is metabolically closer to the normal tissue in EAC patients than to the normal tissue in the controls. Only 3-hydroxybutyrate was increased (*P* < .05) in the normal tissue of the EAC patients compared with the normal tissue of the Barrett's patients.

### Histologically Normal Tissue Samples from controls (Noncancer Patients) and EAC Patients (Class 1 vs 3)

**Figure 6:**
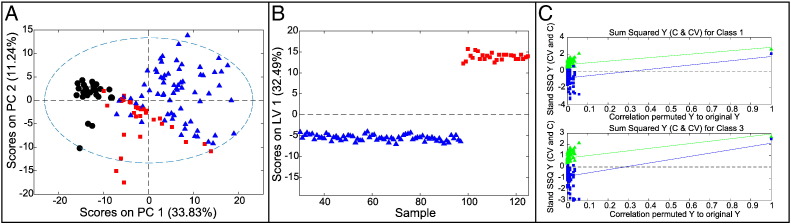
Multivariate models for normal tissues from controls and from EAC patients pre- and postchemotherapy. (A) Scores plot for PCA model comparing normal tissues from controls, EAC patients prechemotherapy, and EAC patients postchemotherapy. Color coding: controls: blue triangles; histologically normal tissue in EAC patients prechemotherapy: red squares; normal tissue in EAC patients postchemotherapy: black circles. (B) Scores plot for PLS-DA model comparing histologically normal tissue from control and EAC patients prechemotherapy. Color coding: controls: blue triangles; EAC patients: red squares. (C) Permutation model for PLS-DA comparing histologically normal tissue from controls and EAC patients prechemotherapy.

Good sample size and significant metabolic differences between the histologically normal tissue samples from controls and EAC patients resulted in a strong PLS-DA model (AUROC = 1.00, CVER = 0.017, *P* = .007, .005; Figure 6), giving a near-perfect separation of histologically normal tissue from controls and EAC patients. This signature arose mainly from 3-hydroxybutyrate, succinate, and formate, which increased (*P* < .0005) along with acetate, glycerophosphocholine, ADP, and lactate (*P* < .05).

These models provide strong evidence of a profound metabolic field effect around EAC tissue.

### Changes Induced by Chemotherapy

Figure S3 shows a scheme for postchemotherapy samples, although the analysis presented here focuses on prechemotherapy samples. It is however noteworthy that the metabolic profile of postchemotherapy samples from normal squamous esophageal mucosa in EAC patients did not shift toward the profile of truly normal squamous esophageal mucosa in control patients (Figure S3). Lactate levels increased postchemotherapy (Figure S3), probably reflecting the Warburg effect in malignant tissues.

## Discussion

Some 211 tissue samples from 121 EAC, BO, or control subjects were examined to identify markers of disease severity and treatment effects. Samples included EAC and BO tissues along with normal squamous epithelia proximal to EAC or BO. The analysis focused on prechemotherapy samples, as chemotherapy itself had a pronounced effect on metabolic signatures.

Previous studies using blood serum or urine have identified various potential biomarkers for EAC and disease progression [13], [15], [22]. However, model power tends to be reduced when the patient groups are more similar. From these studies, it appears that only the extremes can be readily identified from blood samples, and it is possible that the metabolites that have so far been identified may only reflect systemic and therefore advanced disease. From this perspective, tissue samples at or near the site of disease may be more promising. Although there are a few reports for metabolomics studies in esophageal squamous cell carcinoma [23], [24], only two groups have analyzed tissue samples from EAC patients [16], with one study focused on the squamous mucosa of these patients [12]. The results from these studies will be compared with those from our models.

### Field effects in histologically normal squamous tissue

Our study clearly confirms the hypothesis of a field effect observable in metabolite compositions. Metabolomics signatures related to field effects were previously studied by Yakoub et al. [12] who used ^1^H Magic Angle Spinning NMR to compare histologically normal tissue from 35 EAC patients with 52 age-matched controls and observed that a high phosphocholine/glutamate ratio indicated the presence of cancer proximal to histologically normal tissue. In our study, we did observe slightly elevated phosphocholine and indeed glycerophosphocholine, but not reduced glutamate. In our models, the comparison of histologically normal tissue in EAC patients versus controls benefitted from good sample numbers prechemotherapy and gave a strong signature (AUROC = 1) with highly elevated 3-hydroxybutyrate, succinate, and formate and somewhat increased acetate, lactate, ADP, and glycerophosphocholine. As recently suggested by Meiser and Vazquez, the increase in formate may relate to its role in one-carbon metabolism. One possibility is that the reverse activity of cytosolic or mitochondrial 10-formyl-tetrahydrofolate synthetase producing ATP and the by-product, formate from 10-CHO-THF, could be used to produce ATP and NAD(P)H from serine and/or glycine. Thus, the increased levels of cellular formate could be a by-product of ATP and NAD(P)H synthesis as the cells make early changes in how they meet their energy requirements [25].

### Large metabolic differences between squamous and columnar tissues

The present analysis shows that the normal squamous and abnormal columnar tissues have very distinct metabolic signatures and are readily identified in multivariate analyses. Specifically, 3-hydroxybutyrate, hypoxanthine, phosphocholine, and glycerophosphocholine increased, whereas glutamine and alanine decreased. These results are consistent with the early study of Doran et al. which found the choline to creatine ratio increased in EAC tissue compared with proximal normal tissue [16].

These changes can be rationalized as cancer-promoting changes. For example, with cancer cell proliferation, increased amounts of purine nucleotides are required for DNA synthesis, for energy storage in ATP, and for co-factors such as NAD and NADP. In the salvage pathway for purine biosynthesis, hypoxanthine-guanine phosphoribosyl transferase catalyzes the addition of 5-phosphoribose-1-pyrophosphate to hypoxanthine to produce inosine monophosphate. Thus, uptake by cancer cells of hypoxanthine and guanine, produced in other tissues or ingested, should facilitate purine biosynthesis. This rationalizes our observation of steadily increasing levels of hypoxanthine from normal to Barrett's to EAC tissues in EAC patients.

It is well known that 3-hydroxybutyrate levels in blood and tissues may increase during starvation caused by end-stage malignancy. However, in cancer tissue, 3-hydroxybutyrate levels may also be elevated because of the “reverse Warburg effect” whereby energy-rich molecules are taken up by cancer cells perhaps from neighboring cells, are converted to acetyl CoA, and enter the TCA cycle, ultimately generating ATP via oxidative phosphorylation. Evidence supporting this theory comes from the work of Bonuccelli et al. in breast cancer [26]. The fact that, in our models, 3-hydroxybutyrate levels were higher in EAC patients than in controls or Barrett's patients could be a starvation effect. However, the higher 3-hydroxybutyrate in EAC tissue compared with normal tissue in EAC patients is consistent with the “reverse Warburg effect.”

Furthermore, phosphocholine and glycerophosphocholine, components of cell membrane phospholipids, are commonly altered in many cancers, especially in breast cancer [27]. It is therefore not surprising that these metabolites are increased in more proliferative tissues.

Many cancer cells are glutamine dependent in culture as pyruvate gets converted into lactate, and glutaminolysis becomes an important anaplerotic source for the Krebs cycle, thus promoting oxidative phosphorylation and replenishing oxalEACetate lost to anabolism. Also, in certain cancer cells with mitochondrial dysfunction, α-ketoglutarate can be carboxylated to produce isocitrate, citrate, and acetyl-CoA. Thus, the fact that glutamine and indeed glutamate levels are lower in columnar than squamous tissues is perhaps due to greater utilization of glutamine [28].

### Limited metabolic differences among Barrett's and EAC columnar tissues

In the original study of Doran et al., a decrease in the carbohydrate region of the spectrum (3.5-4 ppm) compared with the region around 3 ppm containing resonances from creatine-containing metabolites was observed in Barrett's tissue samples in the presence of EAC compared with Barrett's in the absence of EAC [16]. In our study, creatine levels were decreased, but the result was not statistically significant (*P* > .05). We identified different potential biomarkers for disease progression, including elevated phosphocholine and reduced leucine, valine, and isoleucine by comparing Barrett's tissues in BO and EAC patients. However, all these changes were relatively modest and serve to emphasize that the different types of columnar tissue were much less readily distinguished metabolically.

A larger and longer longitudinal study would be required to validate these markers. The limited differences between Barrett's in Barrett's patients, Barrett's tissue in EAC patients, and EAC tissue in EAC patients are consistent with genetic and epigenetic studies which have indicated that most of the genetic and epigenetic changes present in EAC are also present in Barrett's tissues [29].

## Conclusion

The most interesting finding in the present study was the very strong metabolic signature differentiating normal squamous epithelium from controls and the apparently normal squamous epithelium of EAC patients. Although it is tempting to suggest that this reflects reflux-induced changes in the squamous mucosa of patients with EAC, this seems unlikely because many of the normal control group were referred with dyspeptic symptoms that included reflux. It seems more plausible that the signature is related to a nearby cancer or that the squamous epithelium of the patient destined to develop EAC might have a specific metabolic profile. A metabolic signature for a field effect in histologically normal esophageal squamous mucosa in EAC patients would be of potential diagnostic value to assess risk of progression in adjacent nondysplastic BO at a stage before histologic changes have occurred.

## Conflict of Interest

There is no conflict of interest.

## Funding

This work was supported by a grant by the The Bupa Foundation to R. S. M. R. was in part supported by the COSMOS EU grant (FP7-INFRASTRUCTURES-2012-1-312941) under which all data were deposited to MetaboLights. We thank the Wellcome Trust for support and the Wellcome Building for Biomolecular NMR Spectroscopy where all NMR data have been gathered.

## Figures and Tables

**Table 1:** Tissue Samples Used in This Study

Class	Tissue	Postchemotherapy	Patient Group	Number of Samples
1: NN	Histologically normal squamous esophageal mucosa (“normal tissue”)	N	Controls	68
2: NB	Histologically normal squamous esophageal mucosa (“normal tissue”)	N	Barrett's patients	7
3: NCpr	Histologically normal squamous esophageal mucosa (“normal tissue”)	N	EAC patients	30
4: BB	Nondysplastic Barrett's tissue	N	Barrett's patients	7
5: BCpr	Nondysplastic Barrett's tissue	N	EAC patients	4
6: CCpr	EAC tissue	N	EAC patients	28
7: NCpo	Histologically normal squamous esophageal mucosa (“normal tissue”)	Y	EAC patients	29
8: BCPo	Nondysplastic Barrett's tissue	Y	EAC patients	9
9: CCPo	EAC tissue	Y	EAC patients	29

Abbreviations: First letter indicates tissue type: N: normal tissue. B: Barrett's tissue; C: (cancer) EAC tissue. Second letter indicates patient type: N: normal controls; C: EAC patients; B: Barrett's patients (in absence of EAC). Final two letters: pr: prechemotherapy; po: postchemotherapy.

## Appendix A Supplementary data

**Figure S1**. Boxplots to show metabolite levels measured via peak heights in spectra from 9 tissue types, boxes from left to right are for (1) NN; histologically normal tissue from controls; (2) NB; histologically normal tissue proximal to BO in BO patients; (3) NCpr; histologically normal tissue proximal to OAC in OAC patients, pre-chemotherapy; (4) BB; Barrett’s tissue from BO patients; (5) BCpr; Barrett’s tissue proximal to OAC in OAC patients, pre-chemotherapy; (6) CCpr; OAC tissue in OAC patients, pre-chemotherapy; (7) NCpo; histologically normal tissue proximal to OAC in OAC patients, post-chemotherapy; (8) BCpo; Barrett’s tissue proximal to OAC in OAC patients, post-chemotherapy; (9) CCpo; OAC tissue in OAC patients, post-chemotherapy. Red line=median (not mean); blue box=interquartile range; whiskers=all data not considered to be outliers; red cross=outliers (outliers is more than 1.5 times the interquartile range away from top or bottom of the box, by default). **Figure S2**. Multivariate analysis of the effect of chemotherapy a) Scores plot for PCA model for OAC tissue in OAC patients, pre- and post-chemotherapy b) Scores plot for PLS-DA model for OAC tissue in OAC patients, pre- and post-chemotherapy c) Permutation test n=100 for cancer tissue PLS-DA model, pre- and post-chemotherapy d) Scores plot for PCA model for normal tissue in OAC patients, pre- and post-chemotherapy e) Scores plot for PLS-DA model for normal tissue in OAC patients, pre- and post-chemotherapy f) Permutation test n=100 for normal tissue in OAC patients, pre- and post-chemotherapy Pre-chemotherapy: blue triangles, post-chemotherapy: red squares **Figure S3**. Effect of chemotherapy * denotes p<0.005 ** denotes p<0.0005 **Figure S4**. The Effect of chemotherapy on the PCA and PLS-DA models to separate histologically normal, Barrett’s and OAC tissues in OAC patients. In the PCA model, there was a clear effect of chemotherapy causing an increase in the score in PC2. However, in the PLS-DA model, the only trend observed was that normal tissues, but not other tissue types, separated to some degree in LV1 according to chemotherapy status. The PLS-DA model did not appear to be overly distorted by chemotherapy. a) Scores plot for PCA model* b) Scores plot for PLS-DA model* • colour-coding as follow: brown diamonds and dull blue triangles = post- and pre-chemo normal tissue, respectively; green squares and pink stars=post- and pre-chemo Barrett’s tissue, respectively; brighter blue triangles and white circles= post- and pre-chemo cancer tissue, respectively. Red lines link same tissue type in same patient pre- and post-chemo **Table S1**. Class Comparisons with Parameters for Multivariate Models and Parameters from Univariate Analyses of Individual Metabolite Level Changes. (Mww=Mann-Whitney-Wilcoxon test) Image 1
