# Supplementary material for: Metabolomic Evidence for a Field Effect in Histologically Normal and Metaplastic Tissues in Patients with Esophageal Adenocarcinoma
Source: Neoplasia. 2017 Jan 31;19(3):165–74. doi: 10.1016/j.neo.2016.11.003 (PMC5288314; doi:10.1016/j.neo.2016.11.003)
Supplement: Supplementary file 1 — Figure S1. Boxplots to show metabolite levels measured via peak heights in spectra from 9 tissue types, boxes from left to right are for (1) NN; histologically normal tissue from controls; (2) NB; histologically normal tissue proximal to BO in BO patients; (3) NCpr; histologically normal tissue proximal to OAC in OAC patients, pre-chemotherapy; (4) BB; Barrett’s tissue from BO patients; (5) BCpr; Barrett’s tissue proximal to OAC in OAC patients, pre-chemotherapy; (6) CCpr; OAC tissue in OAC patients, pre-chemotherapy; (7) NCpo; histologically normal tissue proximal to OAC in OAC patients, post-chemotherapy; (8) BCpo; Barrett’s tissue proximal to OAC in OAC patients, post-chemotherapy; (9) CCpo; OAC tissue in OAC patients, post-chemotherapy. Red line=median (not mean); blue box=interquartile range; whiskers=all data not considered to be outliers; red cross=outliers (outliers is more than 1.5 times the interquartile range away from top or bottom of the box, by default). Figure S2. Multivariate analysis of the effect of chemotherapy a) Scores plot for PCA model for OAC tissue in OAC patients, pre- and post-chemotherapy b) Scores plot for PLS-DA model for OAC tissue in OAC patients, pre- and post-chemotherapy c) Permutation test n=100 for cancer tissue PLS-DA model, pre- and post-chemotherapy d) Scores plot for PCA model for normal tissue in OAC patients, pre- and post-chemotherapy e) Scores plot for PLS-DA model for normal tissue in OAC patients, pre- and post-chemotherapy f) Permutation test n=100 for normal tissue in OAC patients, pre- and post-chemotherapy Pre-chemotherapy: blue triangles, post-chemotherapy: red squares Figure S3. Effect of chemotherapy * denotes p<0.005 ** denotes p<0.0005 Figure S4. The Effect of chemotherapy on the PCA and PLS-DA models to separate histologically normal, Barrett’s and OAC tissues in OAC patients. In the PCA model, there was a clear effect of chemotherapy causing an increase in the score in PC2. However, in the PLS-DA model, the o [file mmc1.pdf]

**Table S1: Models and statistical parameters used.**								
Model: Histologically normal tissue, from Barretts patients compared with controls ie Class 2 vs Class 1								
o-PLSDA Class 2 (7) vs Class 1 (68) : 1 Latent variable; ROC=0.94,CVER=0.10, p=0.18 (class 1),0.08 (class 2)								
Metabolite		test	p-value	Mean 1	Mean 2	Std 1	Std 2	Fold change
For	**	Wilcoxon	1.90E-05	1.38E+06	4.93E+06	4.16E+05	2.12E+06	3.6
Lac		Wilcoxon	4.60E-02	5.85E+06	8.21E+06	1.70E+06	4.11E+06	1.4
Model: Barretts tissue compared with histologically normal tissue in Barretts patients ie Class 4 vs Class 2								
ML-PLSDA Class 4 (7) vs Class 2 (7) : ROC=1.00, CVER=0.00, p=0.005								
Metabolite		test	p-value	Mean 1	Mean 2	Std 1	Std 2	Fold change
PCho		paired t-test	1.20E-02	2.97E+06	1.42E+07	9.80E+05	7.76E+06	4.8
Myo		paired t-test	9.40E-03	4.32E+05	1.98E+06	1.73E+05	1.10E+06	4.6
Hpox	*	paired t-test	1.40E-03	2.01E+05	6.29E+05	1.43E+05	2.31E+05	3.1
3HB		paired t-test	2.10E-02	1.18E+05	3.09E+05	4.14E+04	1.72E+05	2.6
glut		paired t-test	6.10E-03	1.62E+06	1.26E+06	4.46E+05	3.82E+05	0.8
Asp		paired t-test	3.70E-02	8.82E+05	7.07E+05	2.80E+05	2.42E+05	0.8
Fum		paired t-test	3.80E-02	4.76E+05	3.66E+05	1.90E+05	1.58E+05	0.8
Lac		paired t-test	5.00E-02	8.21E+06	5.54E+06	4.11E+06	2.35E+06	0.7
Gln	*	paired t-test	1.10E-03	1.31E+06	7.79E+05	2.97E+05	3.51E+05	0.6
ADP		paired Wilcoxon	1.60E-02	6.01E+05	3.89E+05	1.67E+05	1.12E+05	0.6
Ala		paired t-test	5.50E-03	8.13E+06	4.33E+06	2.58E+06	1.55E+06	0.5
Model: Histologically normal tissue from OAC patients (pre-chemotherapy) compared with controls ie Class 3 vs Class 1								
o-PLSDA Class 3 (30) vs Class 1 (68) : ROC=1.00, CVER=0.017, p=0.007 (class 1) ,0.005 (class 3)								
Metabolite		test	p-value	Mean 1	Mean 2	Std 1	Std 2	Fold change
For	**	Wilcoxon	3.90E-15	1.38E+06	5.87E+06	4.16E+05	2.54E+06	4.2
3HB	**	Wilcoxon	7.80E-05	1.62E+05	2.85E+05	1.04E+05	1.64E+05	1.8
Succ	**	Wilcoxon	2.90E-04	9.68E+05	1.55E+06	3.98E+05	1.41E+06	1.6
GPC	*	Wilcoxon	3.90E-03	2.38E+06	3.50E+06	5.18E+05	3.01E+06	1.5
Ace		Wilcoxon	1.40E-02	7.03E+06	9.24E+06	3.04E+06	4.84E+06	1.3
Lac	*	Wilcoxon	4.10E-03	5.85E+06	7.53E+06	1.70E+06	3.16E+06	1.3
ADP		welch test	2.10E-02	4.81E+05	5.90E+05	1.53E+05	2.28E+05	1.2
Asn		Wilcoxon	2.40E-02	2.10E+04	1.67E+04	8.87E+03	1.09E+04	0.8
Model: Barretts tissue compared with histologically normal tissue in OAC patients (pre-chemotherapy) ie Class 5 vs Class 3								
-								
Metabolite		test	p-value	Mean 1	Mean 2	Std 1	Std 2	Fold change
PCho		Wilcoxon	6.90E-03	5.79E+06	2.43E+07	6.88E+06	4.79E+06	4.2
Myo		Wilcoxon	1.30E-02	7.75E+05	1.93E+06	8.09E+05	4.68E+05	2.5
GPC		Wilcoxon	8.10E-03	3.50E+06	5.33E+06	3.01E+06	1.04E+06	1.5
glut	**	welch test	4.60E-04	1.63E+06	1.09E+06	4.59E+05	1.56E+05	0.7
Val	*	welch test	2.60E-03	9.86E+05	6.08E+05	2.63E+05	1.32E+05	0.6
Ala	**	welch test	1.90E-04	6.71E+06	3.71E+06	2.53E+06	7.81E+05	0.6
Asp	*	welch test	5.40E-04	6.43E+05	3.89E+05	2.24E+05	7.48E+04	0.6
Lac		welch test	3.30E-02	7.53E+06	4.88E+06	3.16E+06	1.60E+06	0.6
Gln	**	welch test	1.00E-04	1.49E+06	6.21E+05	5.48E+05	2.06E+05	0.4
Model: Barretts tissue in OAC patients (pre-chemotherapy) compared with Barretts tissue in Barretts patients ie Class 5 vs Class 4								

o-PLSDA Class 5 (4) vs Classs 4 (7) Cross-validation=leave one out : ROC=0.79, CVER=0.27, p=0.332, 0.464								
Metabolite		test	p-value	Mean 1	Mean 2	Std 1	Std 2	Fold change
Ile		welch test	2.70E-02	3.04E+05	2.23E+05	5.72E+04	4.24E+04	0.7
Leu		Wilcoxon	4.20E-02	1.04E+06	6.06E+05	4.64E+05	7.83E+04	0.6
Val		welch test	1.80E-02	1.02E+06	6.08E+05	3.28E+05	1.32E+05	0.6
Asp		welch test	1.30E-02	7.07E+05	3.89E+05	2.42E+05	7.48E+04	0.5
Model: OAC tissue compared with Barretts tissue in OAC patients (pre-chemotherapy) ie Class 6 vs Class 5								
-								
Metabolite		test	p-value	Mean 1	Mean 2	Std 1	Std 2	Fold change
Leu		Wilcoxon	2.40E-02	6.06E+05	1.02E+06	7.83E+04	5.17E+05	1.7
Val		welch test	8.50E-03	6.08E+05	9.04E+05	1.32E+05	2.92E+05	1.5
Ile		Wilcoxon	4.30E-02	2.23E+05	3.36E+05	4.24E+04	1.34E+05	1.5
Asp		welch test	2.20E-02	3.89E+05	5.50E+05	7.48E+04	2.79E+05	1.4
glut		welch test	1.40E-02	1.09E+06	1.42E+06	1.56E+05	4.43E+05	1.3
Gly		welch test	9.00E-03	4.85E+06	6.24E+06	5.22E+05	2.15E+06	1.3
PCho		Wilcoxon	2.40E-02	2.43E+07	1.43E+07	4.79E+06	9.02E+06	0.6
Cr		Wilcoxon	4.30E-02	7.06E+06	4.38E+06	2.13E+06	2.42E+06	0.6
Model: Barretts tissue compared with histologically normal tissue in OAC patients (post-chemotherapy) ie Class 8 vs Class 7								
-								
Metabolite		test	p-value	Mean 1	Mean 2	Std 1	Std 2	Fold change
PCho	**	Wilcoxon	2.80E-05	4.60E+06	1.80E+07	4.84E+06	6.36E+06	3.9
Hpox	*	Wilcoxon	2.00E-03	3.07E+05	4.53E+05	1.71E+05	1.13E+05	1.5
GPC	*	Wilcoxon	7.70E-04	4.30E+06	6.12E+06	3.33E+06	2.56E+06	1.4
ino		Wilcoxon	2.60E-02	1.75E+05	2.30E+05	8.05E+04	5.85E+04	1.3
Gly		welch test	2.40E-02	8.91E+06	7.65E+06	2.36E+06	9.27E+05	0.9
Val		Wilcoxon	1.60E-02	1.30E+06	1.09E+06	2.62E+05	2.00E+05	0.8
Lac		welch test	2.30E-02	1.26E+07	1.07E+07	2.34E+06	1.91E+06	0.8
glut	*	Wilcoxon	2.00E-03	1.40E+06	9.51E+05	3.74E+05	2.21E+05	0.7
Gln		Wilcoxon	6.00E-03	1.42E+06	9.23E+05	3.76E+05	3.53E+05	0.7
Cr	*	welch test	2.00E-03	9.01E+06	6.48E+06	1.76E+06	1.73E+06	0.7
Ala	**	welch test	3.10E-08	8.06E+06	4.82E+06	2.19E+06	6.63E+05	0.6
Succ	**	welch test	3.70E-07	3.90E+06	2.40E+06	1.07E+06	3.99E+05	0.6
Fum	**	welch test	4.50E-04	3.56E+05	2.05E+05	1.34E+05	8.14E+04	0.6
ADP		welch test	6.70E-03	6.01E+05	3.63E+05	2.25E+05	1.91E+05	0.6
Model: OAC tissue compared with Barretts tissue in OAC patients (post-chemotherapy) ie Class 9 vs Class 8								
-								
Metabolite		test	p-value	Mean 1	Mean 2	Std 1	Std 2	Fold change
Asn		Wilcoxon	1.20E-02	1.30E+04	3.10E+04	2.17E+04	2.22E+04	2.4
Hpox		welch test	2.40E-02	4.53E+05	6.22E+05	1.13E+05	3.26E+05	1.4
glut	*	welch test	4.70E-03	9.51E+05	1.26E+06	2.21E+05	3.50E+05	1.3
Myo		Wilcoxon	4.60E-02	2.47E+06	1.99E+06	7.03E+05	1.03E+06	0.8
Cr		Wilcoxon	2.30E-02	6.48E+06	5.11E+06	1.73E+06	3.08E+06	0.8
PCho		Wilcoxon	4.30E-02	1.80E+07	1.28E+07	6.36E+06	8.34E+06	0.7
Model: Histologically normal tissue in OAC patients (pre-chemotherapy) compared with histologically normal tissue in Barretts patients ie Class 3 vs Class 2								
o-PLSDA Class 3 (30) vs Class 2 (7) : dreadful model, 3 LVs ROC=0.70, CVER=0.28, p=0.30,0.30								
Metabolite		test	p-value	Mean 1	Mean 2	Std 1	Std 2	Fold change

3HB	*	Wilcoxon	3.90E-03	1.18E+05	2.85E+05	4.14E+04	1.64E+05	2.4
Model: OAC tissue in OAC patients (pre-chemotherapy) compared with histologically normal tissue in controls ie Class 6 vs Class 1								
o-PLSDA Class 6 (27) vs Class 1 (68) : 1 LV, ROC=1.00, CVER=0.00, p=0.008 (class 6), 0.005 (class 1)								
Metabolite		test	p-value	Mean 1	Mean 2	Std 1	Std 2	Fold change
PCho	**	Wilcoxon	6.80E-10	2.94E+06	1.43E+07	7.67E+05	9.02E+06	4.9
For	**	Wilcoxon	4.70E-13	1.38E+06	5.31E+06	4.16E+05	2.26E+06	3.8
3HB	**	Wilcoxon	2.30E-10	1.62E+05	5.19E+05	1.04E+05	3.75E+05	3.2
GPC	**	Wilcoxon	2.70E-10	2.38E+06	7.72E+06	5.18E+05	5.23E+06	3.2
Hpox	**	Wilcoxon	2.00E-09	2.34E+05	6.18E+05	1.62E+05	2.95E+05	2.6
ino	**	Wilcoxon	2.20E-07	9.26E+04	2.08E+05	9.13E+04	9.21E+04	2.3
Myo	**	Wilcoxon	7.80E-08	5.79E+05	1.28E+06	4.34E+05	5.53E+05	2.2
Succ	*	Wilcoxon	7.60E-04	9.68E+05	1.56E+06	3.98E+05	1.51E+06	1.6
Leu		Wilcoxon	6.80E-03	7.57E+05	1.02E+06	3.18E+05	5.17E+05	1.3
Ile		Wilcoxon	1.10E-02	2.67E+05	3.36E+05	9.45E+04	1.34E+05	1.3
Ace		Wilcoxon	1.20E-02	7.03E+06	6.07E+06	3.04E+06	4.09E+06	0.9
glut		welch test	3.70E-02	1.62E+06	1.42E+06	3.56E+05	4.43E+05	0.9
Asp		welch test	3.00E-02	6.93E+05	5.50E+05	2.98E+05	2.79E+05	0.8
Fum	**	Wilcoxon	2.20E-05	3.95E+05	2.67E+05	1.43E+05	1.00E+05	0.7
ADP	**	Wilcoxon	6.20E-06	4.81E+05	3.16E+05	1.53E+05	1.53E+05	0.7
Ala	**	welch test	8.60E-14	6.02E+06	3.64E+06	1.56E+06	9.67E+05	0.6
Cr	**	Wilcoxon	9.30E-08	7.01E+06	4.38E+06	1.54E+06	2.42E+06	0.6
Gln	**	welch test	4.00E-17	1.44E+06	7.09E+05	3.48E+05	2.61E+05	0.5
Model: OAC tissue compared with histologically normal tissue in OAC patients (pre-chemotherapy) ie Class 6 vs Class 3								
o-PLSDA Class 6 (27) vs Class 1 (68) : 1 LV, ROC=1.00, CVER=0.00, p=0.008 (class 6), 0.005 (class 1)								
Metabolite		test	p-value	Mean 1	Mean 2	Std 1	Std 2	Fold change
PCho	**	Wilcoxon	7.00E-05	5.79E+06	1.43E+07	6.88E+06	9.02E+06	2.5
Hpox	**	Wilcoxon	1.50E-06	2.69E+05	6.18E+05	1.60E+05	2.95E+05	2.3
GPC	**	Wilcoxon	1.80E-05	3.50E+06	7.72E+06	3.01E+06	5.23E+06	2.2
3HB	*	Wilcoxon	1.20E-03	2.85E+05	5.19E+05	1.64E+05	3.75E+05	1.8
ino	*	Wilcoxon	5.70E-04	1.22E+05	2.08E+05	1.14E+05	9.21E+04	1.7
Myo	**	Wilcoxon	2.50E-04	7.75E+05	1.28E+06	8.09E+05	5.53E+05	1.6
Gly		Wilcoxon	1.10E-02	4.95E+06	6.24E+06	1.94E+06	2.15E+06	1.3
Pro		Wilcoxon	1.60E-02	7.32E+05	5.83E+05	5.97E+05	7.97E+05	0.8
Lac		welch test	1.20E-02	7.53E+06	5.78E+06	3.16E+06	1.79E+06	0.8
Ace	**	Wilcoxon	2.50E-04	9.24E+06	6.07E+06	4.84E+06	4.09E+06	0.7
Fum	*	Wilcoxon	2.70E-03	3.84E+05	2.67E+05	1.70E+05	1.00E+05	0.7
Cr	**	Wilcoxon	1.50E-05	6.82E+06	4.38E+06	1.72E+06	2.42E+06	0.6
Ala	**	welch test	3.20E-07	6.71E+06	3.64E+06	2.53E+06	9.67E+05	0.5
Gln	**	welch test	1.30E-08	1.49E+06	7.09E+05	5.48E+05	2.61E+05	0.5
ADP	**	Wilcoxon	5.70E-06	5.90E+05	3.16E+05	2.28E+05	1.53E+05	0.5
Model: Histologically normal tissue in OAC patients, post-chemotherapy vs pre-chemotherapy ie Class 7 vs Class 3								
o-PLSDA Class 7 (29) vs Class 3 (27) : 1 LV, ROC=1.00, CVER=0.00, p=0.005,0.005								
Metabolite		test	p-value	Mean 1	Mean 2	Std 1	Std 2	Fold change
Myo	**	Wilcoxon	3.10E-07	7.75E+05	2.00E+06	8.09E+05	7.62E+05	2.6
Succ	**	Wilcoxon	2.90E-09	1.55E+06	3.90E+06	1.41E+06	1.07E+06	2.5
3HB		Wilcoxon	4.60E-02	2.85E+05	6.42E+05	1.64E+05	6.48E+05	2.3

Gly	**	Wilcoxon	3.90E-08	4.95E+06	8.91E+06	1.94E+06	2.36E+06	1.8
Lac	**	welch test	3.50E-09	7.53E+06	1.26E+07	3.16E+06	2.34E+06	1.7
Asn		Wilcoxon	2.00E-02	1.67E+04	2.40E+04	1.09E+04	1.44E+04	1.4
ino	*	Wilcoxon	2.50E-03	1.22E+05	1.75E+05	1.14E+05	8.05E+04	1.4
Leu	*	welch test	2.50E-03	7.83E+05	1.01E+06	2.55E+05	3.01E+05	1.3
Val	**	welch test	2.50E-05	9.86E+05	1.30E+06	2.63E+05	2.62E+05	1.3
Ile	*	Wilcoxon	8.70E-04	2.77E+05	3.72E+05	9.32E+04	1.13E+05	1.3
Cr	**	welch test	1.10E-05	6.82E+06	9.01E+06	1.72E+06	1.76E+06	1.3
Tyr	**	welch test	6.00E-05	2.16E+05	2.74E+05	4.47E+04	5.71E+04	1.3
Ala		welch test	3.20E-02	6.71E+06	8.06E+06	2.53E+06	2.19E+06	1.2
GPC	**	Wilcoxon	1.80E-04	3.50E+06	4.30E+06	3.01E+06	3.33E+06	1.2
Tau		welch test	1.10E-02	6.98E+06	8.18E+06	1.80E+06	1.69E+06	1.2
Ace	**	Wilcoxon	6.00E-06	9.24E+06	4.91E+06	4.84E+06	1.98E+06	0.5
Pro	**	Wilcoxon	6.10E-09	7.32E+05	2.58E+05	5.97E+05	1.40E+05	0.4
Asp	**	Wilcoxon	5.10E-09	6.43E+05	2.82E+05	2.24E+05	1.11E+05	0.4
For	**	Wilcoxon	2.70E-10	5.87E+06	1.31E+06	2.54E+06	9.16E+05	0.2
Model: OAC tissue in OAC patients, post-chemotherapy vs pre-chemotherapy ie Class 9 vs Class 6								
o-PLSDA Class 9 (28) vs Class 6 (27) : 2 LVs, ROC=1.00, CVER=0.018,p=0.007,0.007								
Metabolite		test	p-value	Mean 1	Mean 2	Std 1	Std 2	Fold change
Lac	**	welch test	4.40E-12	5.78E+06	1.21E+07	1.79E+06	3.13E+06	2.1
Ile	**	Wilcoxon	5.60E-05	3.36E+05	5.36E+05	1.34E+05	2.60E+05	1.6
Succ	**	Wilcoxon	8.80E-06	1.56E+06	2.44E+06	1.51E+06	8.38E+05	1.6
Myo	*	Wilcoxon	4.00E-03	1.28E+06	1.99E+06	5.53E+05	1.03E+06	1.6
Asn		Wilcoxon	3.40E-02	2.05E+04	3.10E+04	1.12E+04	2.22E+04	1.5
Tyr		Wilcoxon	7.50E-03	2.07E+05	2.95E+05	8.73E+04	1.51E+05	1.4
Leu	*	Wilcoxon	3.80E-03	1.02E+06	1.33E+06	5.17E+05	6.36E+05	1.3
Val	*	Wilcoxon	3.10E-03	9.04E+05	1.21E+06	2.92E+05	4.45E+05	1.3
Ala	*	welch test	1.90E-03	3.64E+06	4.59E+06	9.67E+05	1.21E+06	1.3
Gly	*	welch test	6.20E-04	6.24E+06	8.24E+06	2.15E+06	2.00E+06	1.3
Ace	*	Wilcoxon	2.80E-03	6.07E+06	4.15E+06	4.09E+06	1.72E+06	0.7
Fum	*	Wilcoxon	1.00E-03	2.67E+05	1.86E+05	1.00E+05	1.05E+05	0.7
Asp	**	Wilcoxon	2.60E-04	5.50E+05	3.35E+05	2.79E+05	2.02E+05	0.6
Pro	**	Wilcoxon	3.80E-06	5.83E+05	2.63E+05	7.97E+05	2.70E+05	0.5
For	**	Wilcoxon	1.20E-09	5.31E+06	1.28E+06	2.26E+06	8.05E+05	0.2
Model: Histologically normal tissue vs OAC tissue in OAC patients, post-chemotherapy ie Class 9 vs Class 7								
o-PLSDA Class 9 (28) vs Class 7 (29) : 1 LV, ROC=0.94, CVER=0.14, p=0.023 (Class 7),0.28 (Class 9)								
Metabolite		test	p-value	Mean 1	Mean 2	Std 1	Std 2	Fold change
PCho	**	Wilcoxon	7.50E-06	4.60E+06	1.28E+07	4.84E+06	8.34E+06	2.8
Hpox	**	Wilcoxon	9.30E-06	3.07E+05	6.22E+05	1.71E+05	3.26E+05	2
GPC	**	Wilcoxon	8.10E-08	4.30E+06	7.68E+06	3.33E+06	3.31E+06	1.8
ino		Wilcoxon	1.10E-02	1.75E+05	2.55E+05	8.05E+04	1.42E+05	1.5
Ile	*	Wilcoxon	1.40E-03	3.72E+05	5.36E+05	1.13E+05	2.60E+05	1.4
Leu		Wilcoxon	2.40E-02	1.01E+06	1.33E+06	3.01E+05	6.36E+05	1.3
Asn		Wilcoxon	3.70E-02	2.40E+04	3.10E+04	1.44E+04	2.22E+04	1.3
Ala	**	welch test	2.40E-09	8.06E+06	4.59E+06	2.19E+06	1.21E+06	0.6
Succ	**	welch test	4.40E-07	3.90E+06	2.44E+06	1.07E+06	8.38E+05	0.6
Cr	**	Wilcoxon	1.40E-07	9.01E+06	5.11E+06	1.76E+06	3.08E+06	0.6
Gln	**	Wilcoxon	1.40E-07	1.42E+06	7.61E+05	3.76E+05	2.70E+05	0.5
Fum	**	Wilcoxon	2.30E-06	3.56E+05	1.86E+05	1.34E+05	1.05E+05	0.5
ADP	**	Wilcoxon	1.20E-06	6.01E+05	2.73E+05	2.25E+05	1.72E+05	0.5

Model: 10 case model: Barretts vs histologically normal tissues in OAC patients								
ML-PLSDA model : ROC=1.00, CVER=0.00, p=0.00								
Metabolite		test	p-value	Mean 1	Mean 2	Std 1	Std 2	Fold change
PCho	**	paired t-test	6.40E-05	3.46E+06	1.99E+07	6.03E+05	7.43E+06	5.7
ino		paired t-test	1.10E-02	1.35E+05	2.37E+05	5.42E+04	7.13E+04	1.8
Hpox	*	paired t-test	1.20E-03	2.45E+05	4.37E+05	7.51E+04	1.34E+05	1.8
GPC	*	paired Wilcoxon	2.00E-03	3.39E+06	5.68E+06	4.81E+05	2.13E+06	1.7
Myo	*	paired t-test	4.60E-03	1.26E+06	2.19E+06	5.99E+05	5.15E+05	1.7
3HB	*	paired Wilcoxon	2.00E-03	6.34E+05	9.24E+05	7.80E+05	9.48E+05	1.5
Val		paired t-test	1.70E-02	1.21E+06	9.91E+05	2.73E+05	2.98E+05	0.8
Cr		paired t-test	3.70E-02	8.31E+06	6.48E+06	1.92E+06	1.96E+06	0.8
Lac		paired t-test	6.60E-03	1.10E+07	8.76E+06	4.02E+06	3.68E+06	0.8
glut	*	paired t-test	5.90E-04	1.48E+06	1.00E+06	3.74E+05	2.36E+05	0.7
Ala	**	paired t-test	6.40E-06	8.19E+06	4.52E+06	1.51E+06	9.66E+05	0.6
Succ	*	paired t-test	2.70E-03	3.01E+06	1.92E+06	1.49E+06	7.68E+05	0.6
Fum	*	paired Wilcoxon	2.00E-03	3.33E+05	2.11E+05	1.06E+05	8.28E+04	0.6
ADP		paired t-test	6.40E-03	6.52E+05	4.10E+05	2.09E+05	2.25E+05	0.6
Gln	**	paired t-test	1.50E-04	1.81E+06	9.03E+05	2.84E+05	3.47E+05	0.5
Model: 10 case model: OAC vs Barretts tissues in OAC patients								
ML-PLSDA model : ROC=0.85, CVER=0.29, p=0.055								
Metabolite		test	p-value	Mean 1	Mean 2	Std 1	Std 2	Fold change
Asn		paired Wilcoxon	5.90E-03	1.17E+04	2.66E+04	2.10E+04	1.06E+04	2.3
Hpox		paired t-test	5.30E-03	4.37E+05	8.25E+05	1.34E+05	3.30E+05	1.9
GPC		paired Wilcoxon	9.80E-03	5.68E+06	8.99E+06	2.13E+06	3.28E+06	1.6
glut		paired t-test	2.20E-02	1.00E+06	1.34E+06	2.36E+05	2.56E+05	1.3
Cr		paired t-test	2.00E-02	6.48E+06	4.36E+06	1.96E+06	1.20E+06	0.7
Pro		paired Wilcoxon	3.70E-02	4.35E+05	2.48E+05	2.89E+05	1.06E+05	0.6
Ace		paired Wilcoxon	2.70E-02	7.06E+06	4.50E+06	4.52E+06	2.14E+06	0.6
Model: 10 case model: OAC vs normal tissues in OAC patients								
Metabolite		test	p-value	Mean 1	Mean 2	Std 1	Std 2	Fold change
PCho	*	paired t-test	9.80E-04	3.46E+06	1.80E+07	6.03E+05	9.84E+06	5.2
Hpox	**	paired t-test	4.30E-04	2.45E+05	8.25E+05	7.51E+04	3.30E+05	3.4
GPC	**	paired t-test	1.60E-04	3.39E+06	8.99E+06	4.81E+05	3.28E+06	2.7
ino	*	paired t-test	7.20E-04	1.35E+05	2.79E+05	5.42E+04	9.43E+04	2.1
3HB	*	paired Wilcoxon	2.00E-03	6.34E+05	9.23E+05	7.80E+05	1.01E+06	1.5
Fum		paired Wilcoxon	9.80E-03	3.33E+05	2.26E+05	1.06E+05	5.79E+04	0.7
Pro		paired Wilcoxon	2.00E-02	4.03E+05	2.48E+05	3.03E+05	1.06E+05	0.6
Ala	**	paired t-test	2.70E-04	8.19E+06	4.55E+06	1.51E+06	1.44E+06	0.6
Succ	*	paired t-test	4.90E-03	3.01E+06	1.91E+06	1.49E+06	8.24E+05	0.6

Cr	**	paired t-test	4.20E-04	8.31E+06	4.36E+06	1.92E+06	1.20E+06	0.5
Gln	**	paired t-test	1.60E-05	1.81E+06	8.13E+05	2.84E+05	2.16E+05	0.4
ADP	**	paired t-test	3.50E-04	6.52E+05	2.86E+05	2.09E+05	1.10E+05	0.4
10 case model: OAC vs Barretts vs histologically normal tissues in OAC patients								
o-PLSDA model : 2 LVs, for the normal, Barretts and cancer classes respectively ROC=1.00,0.86,0.91 (OAC), CVER=0.00,0.23,0.13, p=0.021,0.14,0.049								

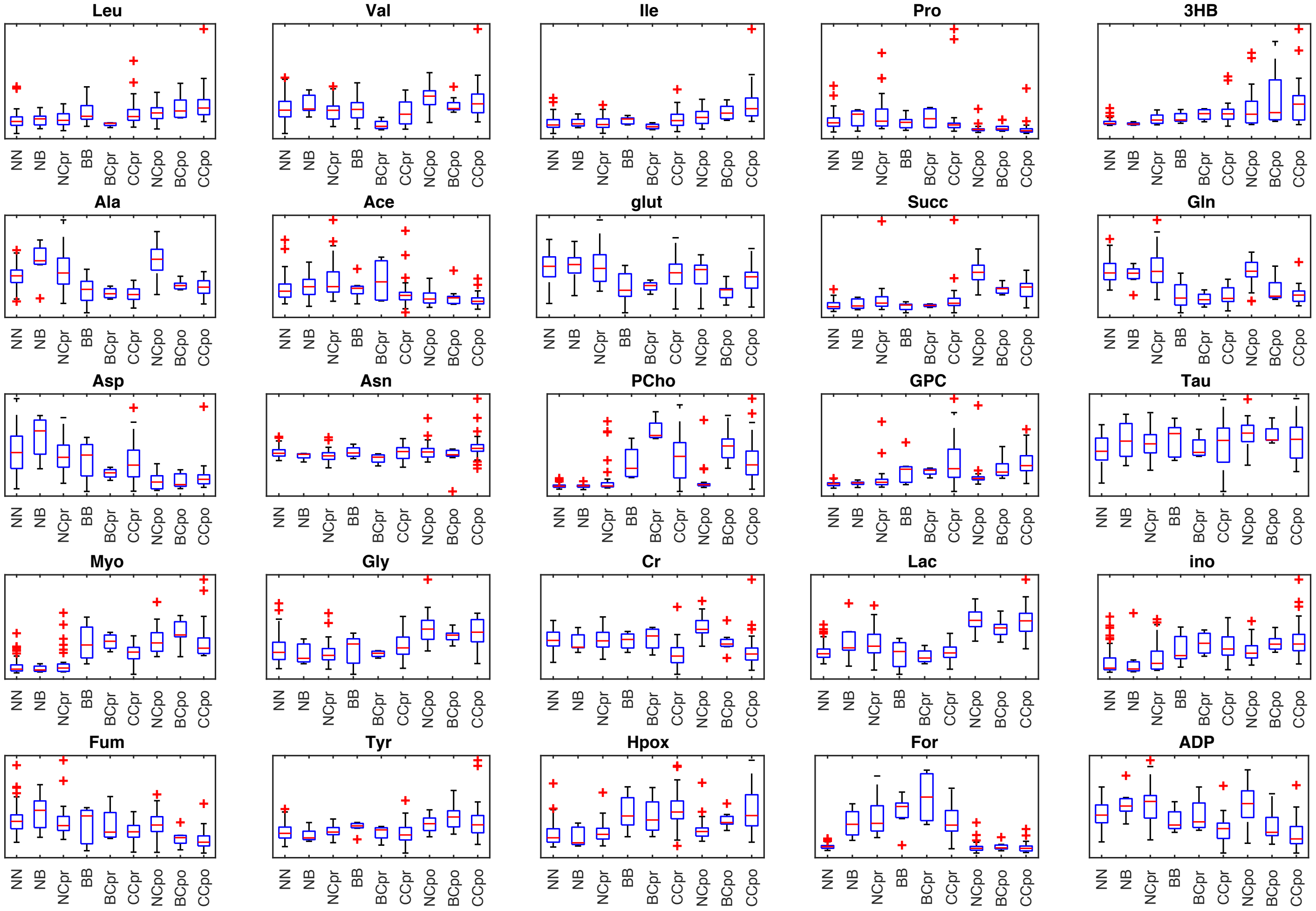

Figure S1. Boxplots to show metabolite levels measured via peak heights in spectra from 9 tissue types, boxes from left to right are for (1) NN; histologically normal tissue from controls; (2) NB; histologically normal tissue proximal to BO in BO patients; (3) NCpr; histologically normal tissue proximal to OAC in OAC patients, pre-chemotherapy; (4) BB; Barrett's tissue from BO patients; (5) BCpr; Barrett's tissue proximal to OAC in OAC patients, pre-chemotherapy; (6) CCpr; OAC tissue in OAC patients, pre-chemotherapy; (7) NCpo; histologically normal tissue proximal to OAC in OAC patients, post-chemotherapy; (8) BCpo; Barrett's tissue proximal to OAC in OAC patients, post-chemotherapy; (9) CCpo; OAC tissue in OAC patients, post-chemotherapy.

Red line=median (not mean); blue box=interquartile range; whiskers=all data not considered to be outliers; red cross=outliers (outliers is more than 1.5 times the interquartile range away from top or bottom of the box, by default).

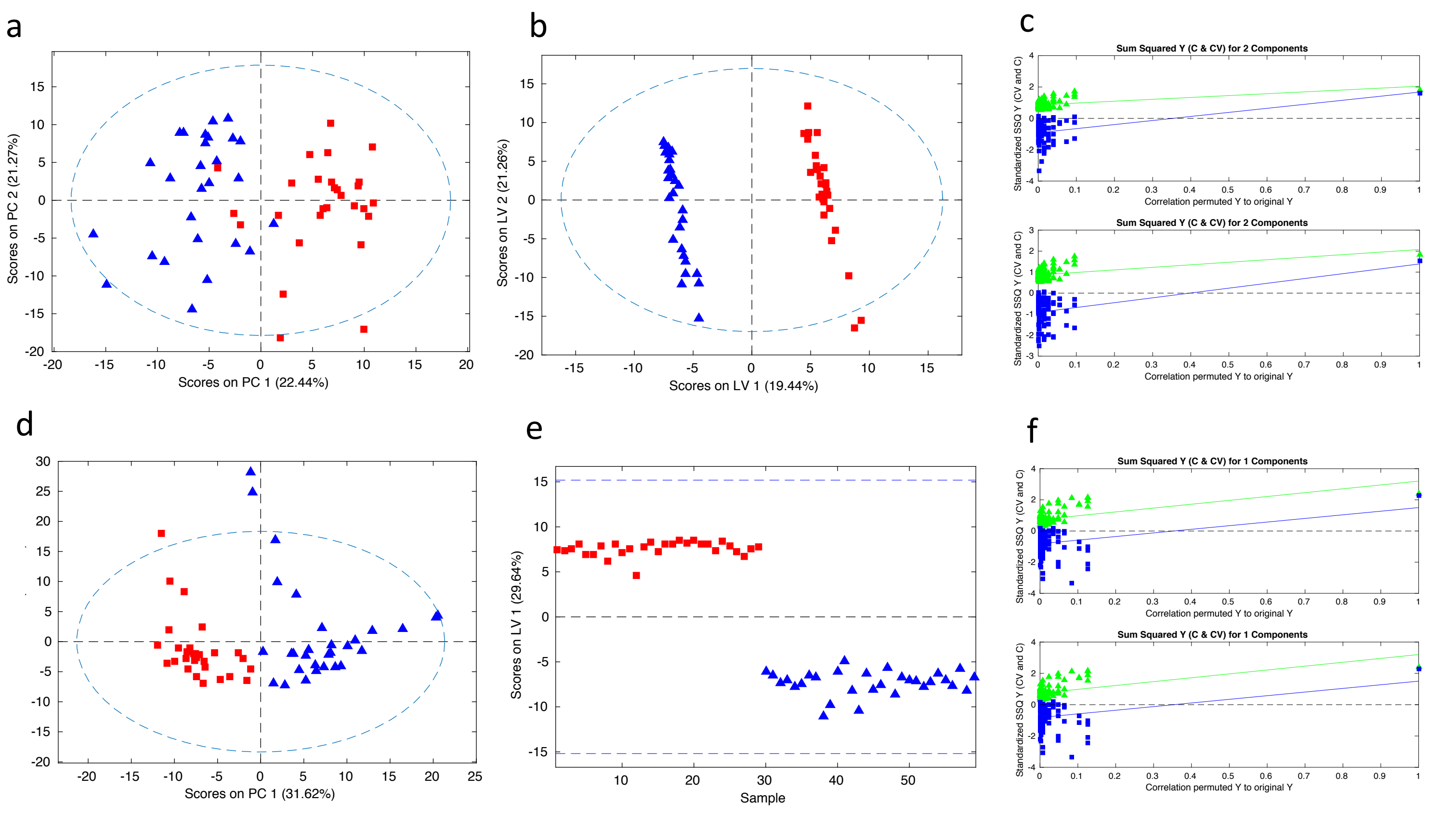

Figure S2: Multivariate analysis of the effect of chemotherapy

a) Scores plot for PCA model for OAC tissue in OAC patients, pre- and post-chemotherapy
b) Scores plot for PLS-DA model for OAC tissue in OAC patients, pre- and post-chemotherapy
c) Permutation test n=100 for cancer tissue PLS-DA model, pre- and post-chemotherapy
d) Scores plot for PCA model for normal tissue in OAC patients, pre- and post-chemotherapy
e) Scores plot for PLS-DA model for normal tissue in OAC patients, pre- and post-chemotherapy
f) Permutation test n=100 for normal tissue in OAC patients, pre- and post-chemotherapy

Pre-chemotherapy: blue triangles, post-chemotherapy: red squares

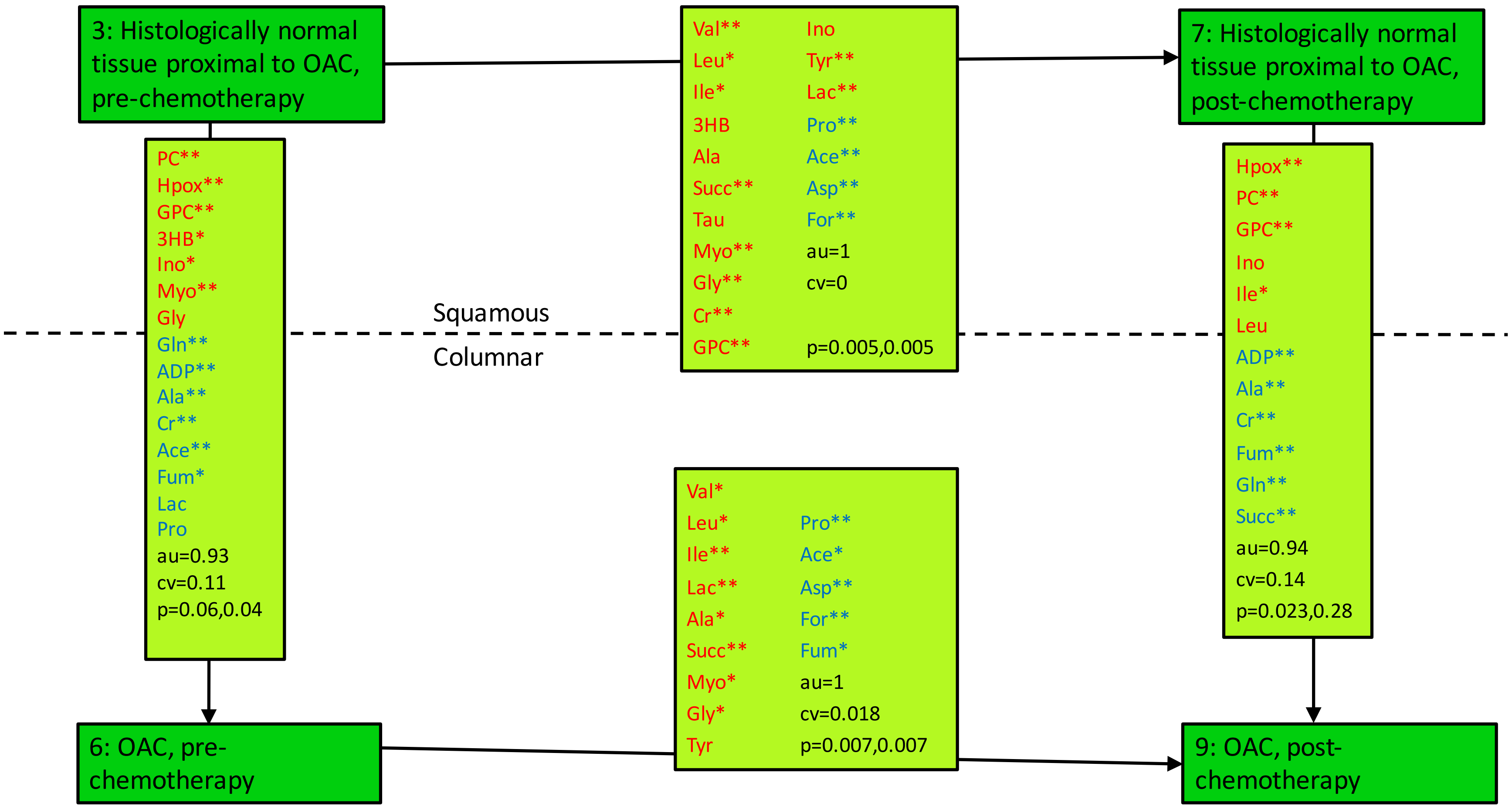

Figure S3: Effect of chemotherapy
* denotes p<0.005
** denotes p<0.0005

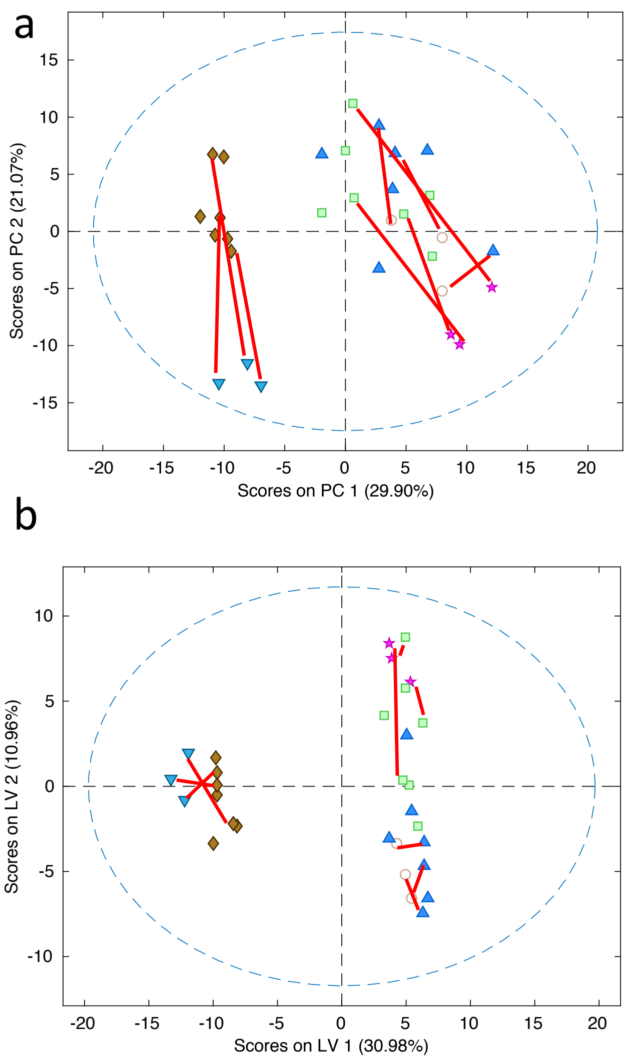

Figure S4:
The Effect of chemotherapy on the PCA and PLS-DA models to separate histologically normal, Barrett's and OAC tissues in OAC patients.
In the PCA model, there was a clear effect of chemotherapy causing an increase in the score in PC2. However, in the PLS-DA model, the only trend observed was that normal tissues, but not other tissue types, separated to some degree in LV1 according to chemotherapy status. The PLS-DA model did not appear to be overly distorted by chemotherapy.
a) Scores plot for PCA model*
b) Scores plot for PLS-DA model*
• colour-coding as follow: brown diamonds and dull blue triangles = post- and pre-chemo normal tissue, respectively; green squares and pink stars=post- and pre-chemo Barrett's tissue, respectively; brighter blue triangles and white circles= post- and pre-chemo cancer tissue, respectively.
Red lines link same tissue type in same patient pre- and post-chemo
